# Reactive lymphoid hyperplasia of the liver: A case report

**DOI:** 10.1097/MD.0000000000016491

**Published:** 2019-07-26

**Authors:** Wenyan Zhang, Shusen Zheng

**Affiliations:** Department of Surgery, Division of Hepatobiliary and Pancreatic Surgery, First Affiliated Hospital, School of Medicine, Zhejiang University, Hangzhou, Zhejiang Province, China.

**Keywords:** differential diagnosis, hepatectomy, liver lesion, reactive lymphoid hyperplasia

## Abstract

**Rationale:**

Reactive lymphoid hyperplasia (RLH) of the liver is an uncommon benign lesion. It is usually difficult to differentiate from hepatocellular carcinoma (HCC), peripheral-type cholangiocellular carcinoma (CCC), combined HCC-CCC, and liver metastases. On account of its rarity and controversial issues, we report a case report and review the literature to discuss its clinical features, treatments, radiological, and immunohistochemical characteristics.

**Patient concerns:**

A 54-year-old woman had a history of primary biliary cirrhosis and chronic cholecystitis. She complained of finding a lesion in the right liver during her last medical check-up by abdominal B type ultrasound. The Contrast-enhanced computed tomography revealed a circular and low-density lesion in the right posterior lobe of the liver, approximately 22.0 × 18.7 mm in size. On magnetic resonance imaging, the lesion showed low-signal intensity on T1-weighted images and high signal intensity on T2-weighted images. Laboratory test results were almost normal.

**Diagnosis:**

After the postoperative pathological and immunohistochemical examination, the patient was finally diagnosed as having RLH.

**Interventions:**

The patient received right posterior lobe hepatectomy and cholecystectomy.

**Outcomes:**

The patient was discharged 11 days after surgery. No evidence of recurrence was noted 1 year after the surgery.

**Lessons:**

Although RLH of the liver is rare, it is necessary to be considered in a liver lesion, especially in female patients. This case report may advance the understanding of RLH of the liver and reduce the number of mistakenly diagnosed patients.

## 1 Introduction

Reactive lymphoid hyperplasia (RLH), also known as pseudolymphoma or nodular lymphoid lesion of the liver was first reported in 1981 by Snover et al.^[1]^ RLH was a benign nonspecific lesion characterized by a marked proliferation of polyclonal lymphocytes forming follicles with an active germinal center.^[2]^ RLH had been reported in various organs, including the skin,^[3]^ lung,^[4]^ eye orbit,^[5]^ intestine,^[6]^ and thyroid,^[7]^ but it is an uncommon benign lesion in the liver. It was usually difficult to differentiate from hepatocellular carcinoma (HCC), peripheral-type cholangiocellular carcinoma (CCC), combined HCC-CCC, and liver metastases. There are only 68 cases have been reported in the English literature. The precise etiology and pathogenesis have not yet been unknown, but it was speculated to represent a reactive immunological response to a chronic infection or inflammatory process^[8]^ and associated with malignant tumor.^[9]^ The diagnosis of RLH of the liver was usually difficult because the imaging findings and laboratory test results were nonspecific. The diagnosis usually had not been made until a pathological and immunohistochemical examination was performed after surgical resection. Herein, we describe a patient with RLH of the liver with right upper abdomen dull pain repeatedly.

## 2 Case presentation

A 54-year-old woman complained of finding a lesion in the right liver during her last medical check-up by abdominal B type ultrasound. Further inquiry revealed recurrent right upper abdomen dull pain during the last few months. She was diagnosed of primary biliary cirrhosis 2 months ago in local hospital and had the history of chronic cholecystitis for >20 years. All physical examination findings were normal. No elevation of tumor markers including α-fetoprotein, carcinoembryonic antigen (CEA), cancer antigen 125, and cancer antigen 199 was observed. Hepatitis virus markers were all negative and liver function test was normal. HCC was suspected. Therefore, abdominal contrast-enhanced computed tomography (CT) scan was arranged to confirm the diagnosis.

**Figure 1:**
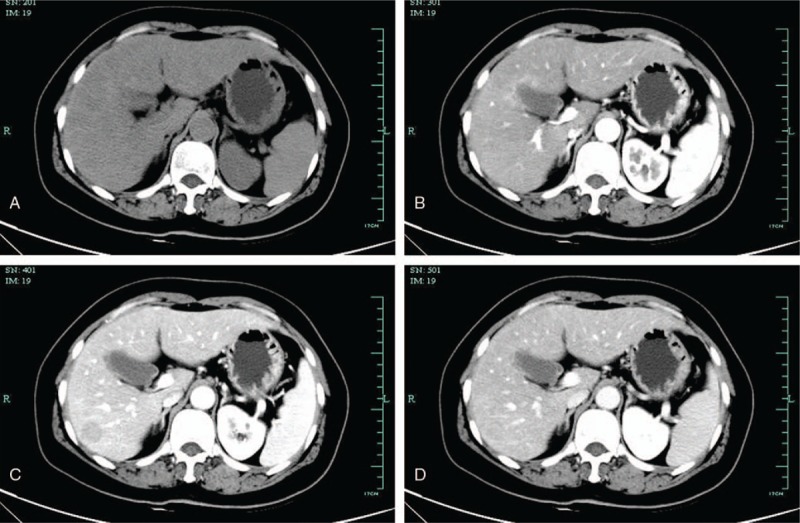
Liver contrast-enhanced computed tomography findings. There was a circular and low-density lesion in the right posterior lobe of the liver, approximately 22.0 × 18.7 mm in size. The lesion showed relatively low density in arterial phase and faded signal in portal phase and delayed phase. (A: plain scanning phase; B: arterial phase; C: portal phase; D: delayed phase).

The CT (Fig. 1) revealed a circular and low density lesion in the right posterior lobe of the liver, approximately 22.0 × 18.7 mm in size. The lesion showed relatively low density in arterial phase and faded signal in portal phase and delayed phase. HCC was highly suspected, and hepatic steatosis and cirrhosis were also noted.

**Figure 2:**
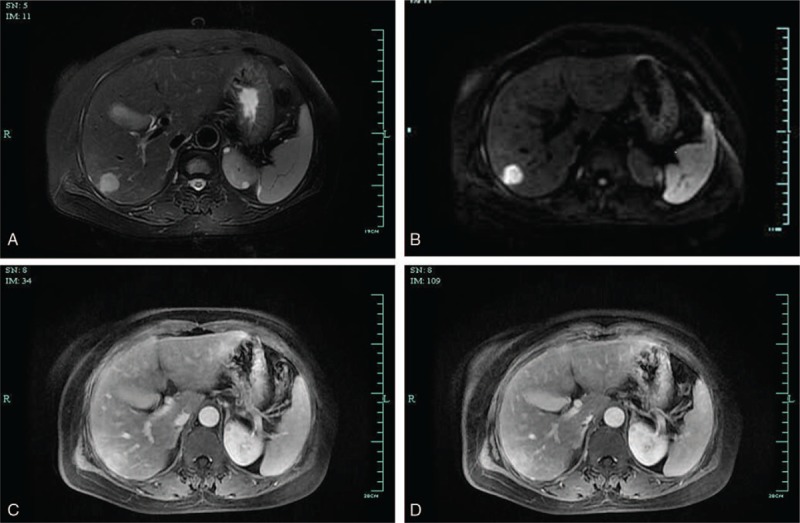
Abdominal magnetic resonance imaging (MRI) findings. The lesion was located in right liver measuring about 20 × 18 mm and showed low SI on T1WI, high signal intensity (SI) on T2-weighted imaging (T2WI) and high SI on diffusion-weighted imaging (DWI). The lesion showed enhancement on arterial phase and faded signal on portal and delayed phases. Hypervascular neoplasm was suspected by MRI. (A: T2WI; B: DWI; C: arterial phase; D: portal venous phase).

The patient was admitted for further examination and treatment. No abnormality was found by blood tests. Abdominal magnetic resonance imaging (MRI) (Fig. 2) showed that the lesion was located in right liver measuring about 20 × 18 mm and showed low signal intensity (SI) on T1-weighted images (T1WI) and high SI on T2-weighted images (T2WI). The lesion showed enhancement on arterial phase and faded signal on portal and delayed phases. Hypervascular neoplasm was suspected according to MRI.

**Figure 3:**
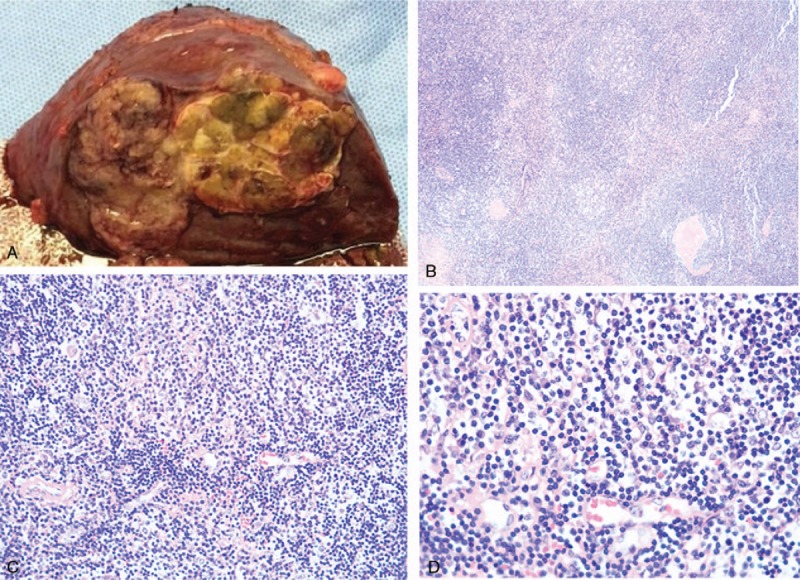
The pathological examination findings. (A) A gray firm lesion about 20 mm in diameter was found in segment VII of the liver. (B–D): Microscopically, there were lymphoid cell proliferation and lymphoid follicles formation in lesion. (B: hematoxylin and eosin [HE] ×100; C: HE ×200; D: HE ×400).

The patient received right posterior hepatectomy and cholecystectomy under the impression of HCC. During the operation, a gray firm lesion about 20 mm in size was found in segment VII of the liver (Fig. 3A). The operation had lasted about 5 hours, and the volume of blood loss was about 200 mL. The pathological examination showed, microscopically, lymphoid cells proliferation and lymphoid follicles formation in lesion (Fig. 3B–D). Immunohistochemical results were as follows: CD3(T+), CD20(B+), Ki-67(nearly 15%), CD10(follicles+), BcL-2(part +), Kappa(K) (a little +), Lambda (λ)(a little +), CD138 (a little +), EBER (−), CD21(FDC+), CD23 (part +), CD35 (FDC+), SMA (−), MUM1 (a little +), CD79a (B+), IGg4 (several+). The diagnosis of RLH was finally confirmed.

The postoperative course was uneventful and no adjuvant therapy, such as chemotherapy, was administered. No evidence of recurrence was noted 1 year after the surgery.

## 3 Discussion

We report the case of a patient with RLH of the liver. The etiology and pathogenesis of RLH of the liver has not yet been unknown, but it has been speculated to represent a reactive immunological response to a chronic infection or inflammatory process.^[8]^ It was first described in the lung by Saltzstein et al in 1963.^[10]^ RLH of the liver is an uncommon benign lesion and was first reported in 1981 by Snover et al.^[1]^ RLH is defined histologically as the aggregation of lymphoid follicles typically with reactive hyperplasia of germinal centers showing proliferation of polyclonal lymphocytes without atypia.^[2]^

We reviewed the published literature until 2018/12/25, using the keywords “liver,” “pseudolymphoma,” and “reactive lymphoid hyperplasia,” and we found 69 cases of RLH of the liver. These cases and our case (70 cases) are summarized in Table 1.

**Table 1:**
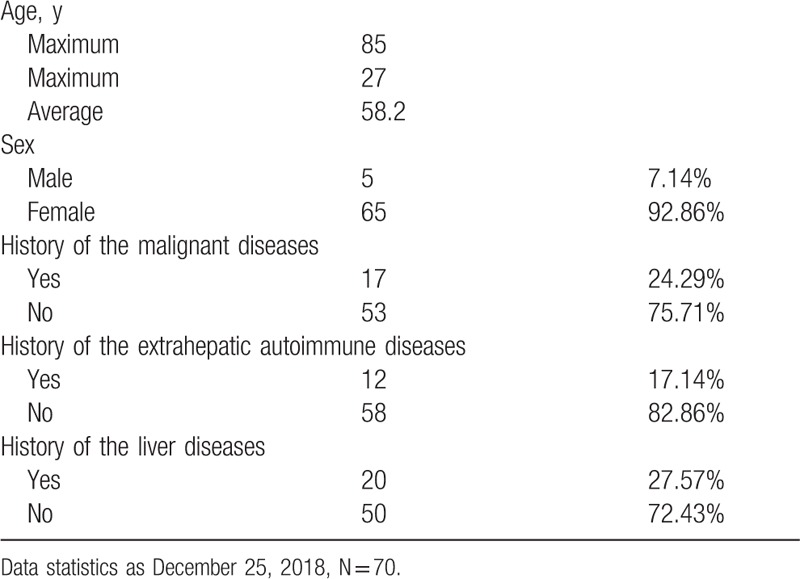
The summary of reactive lymphoid hyperplasia of the liver.

The mean age of the patients was 58.2 years (range, 27–85). Of the 70 cases, 5 (7.14%) were male patients and 65 (92.86%) were female patients. RHL mostly affected women with single lesion ranged from 4 to 55 mm,^[11]^ most <20 mm.^[12]^ In other words, if we found a single and small (≤20 mm) tumor in female patients, the possibility of RLH of the liver should be considered.

Among the 70 cases, 17 (24.29%) had malignant diseases, including thyroid cancer gastric cancer, colon cancer, uterine/ovarian cancer, renal cell carcinoma, pancreatic cancer, common bile duct cancer, and HCC. It had been reported the connection between RLH and malignant tumors.^[9]^ However, this connection might be a result of more regular check-up in patients bearing with tumors, but not the tumors themselves.

Although the etiopathogenesis of RLH of the liver is unknown, RLH of the liver was considered to be resulted from mechanical stimulation in patients with extrahepatic autoimmune diseases.^[13]^ Among the 70 cases reviewed, 12 (17.14%) had extrahepatic autoimmune diseases, including Sjogren syndrome, autoimmune thyroiditis, Takayasu aortitis, antiphospholipid syndrome, and CREST syndrome, among others.

RLH of the liver had been reported to be associated with chronic liver diseases. Among the 70 cases reviewed, 20 (28.57%) had chronic liver diseases, including primary biliary cirrhosis in 12cases, viral hepatitis in 6 cases, and nonalcoholic steatohepatitis in 2 cases. The present patient also had the history of primary biliary cirrhosis. RLH of the liver had been reported to develop after interferon treatment for chronic hepatitis.^[14,15]^ In addition, lymphoid follicles are generally found in a liver with chronic liver diseases but not in a normal liver.^[2]^

Imaging findings of RLH of the liver are similar to malignant lesions of the liver, such as HCC, CCC, and liver metastatic tumor. Therefore, preoperative diagnosis of RLH of the liver is extremely difficult with imaging findings alone. Among the 70 reviewed cases, 29(41.43%) had been misdiagnosed with HCC preoperatively. And, to our best knowledge, there was no case diagnosed with RLH of the liver preoperatively. RLH of the liver tended to show low density on CT, slight early enhancement in arterial phase, and low to isodensity in the delayed phase.^[16]^ However, in this case, it was low-density lesion in arterial phase. The signal faded in portal phase and delayed phase. On MRI, the lesion tended to reveal low SI on T1WI and high SI on T2WI, which was consistent with our case.^[16]^ However, all these imaging findings were not specific for RLH of the liver. According to the reports of CT and MRI in this case, we also misdiagnosed with HCC preoperatively.

Hepatic biopsy had been considered to get a definitive diagnosis. But we did not think it would be able to provide an accurate diagnosis and hepatic biopsy may lead to malignant tumor cells metastasis. Therefore, we decided to perform the right posterior lobe hepatectomy and cholecystectomy rather than hepatic biopsy. Finally, the immunohistochemical results helped us to make the accurate diagnosis. In this respect, the difference between RLH of the liver and other liver malignant tumors, especially HCC, were obvious.

It was reported that malignant transformation of RLH into lymphoma in lung, stomach, and skin existed.^[17–19]^ However, RLH of the liver is a benign lesion, and there are no reports of malignant transformation or local or distant recurrence of RLH of the liver from various follow-up periods ranging from 3 months to 5 years.^[20]^ Thus, if the preoperative diagnosis was established, surgical resection may not be necessary and routine follow-up was recommended.^[21]^ But as the radiological features of RLH was similar to malignant lesions, preoperative diagnosis was often challenging. Therefore, at present stage, surgery operation is still the most best treatment option.

## 4 Conclusions

We herein described a patient with RLH of the liver with recurrent right upper abdomen dull pain. The diagnosis of RLH of the liver was not made until pathological and immunohistochemical examination was performed after surgical resection. This case showed that when a diagnosis of RLH of the liver is temporarily impossible, timely surgical treatment may be helpful for an early diagnosis, thereby improving the prognosis.

## Author contributions

**Conceptualization:** Wenyan Zhang.

**Data curation:** Wenyan Zhang.

**Investigation:** Wenyan Zhang.

**Project administration:** Shusen Zheng.

**Resources:** Wenyan Zhang.

**Writing – original draft:** Wenyan Zhang.

**Writing – review & editing:** Shusen Zheng.
